# 

**Published:** 1863-09

**Authors:** 


New Series, Vol. 6, No. 9. Whole Series, Vol. 20, No. 9.

B04 1305

### THE CHICAGO

### MEDICAL JOURNAL.

DANIEL BRAINARD, M. D.,
J. ADAMS ALLEN, M. D.,
Editors and Proprietors.

SEPTEMBER, 1863.

CHICAGO:

J. C. W. BAILEY, BOOK AND JOB PRINTER, 130 CLARK STREET.

1863.

